# Supplementary material for: Visual aids in ambulatory clinical practice: Experiences, perceptions and needs of patients and healthcare professionals
Source: PLoS One. 2022 Feb 2;17(2):e0263041. doi: 10.1371/journal.pone.0263041 (PMC8809598; doi:10.1371/journal.pone.0263041)
Supplement: Attachment — Submitted filename: Revision letter final 10.04.docx [file pone.0263041.s001.docx]

Dear Editor, Dear Dr Trayler,

We would like to thank you for your careful review of our manuscript. We have addressed our answers to each of the reviewers’ comments one-by-one below and have outlined changes made in the manuscript. We’ve attached two versions of the revised manuscript, one with marked revisions, and one clean version.

We believe the manuscript has now improved in quality and clarity.

We hope this revised version will meet with your approval and remain at your disposal, should you wish any further information.

Best regards,

Catherine Hafner on behalf of the research team

**Editor’s comments :**

1. Please ensure that your manuscript meets PLOS ONE's style requirements, including those for file naming. The PLOS ONE style templates can be found at

<https://journals.plos.org/plosone/s/file?id=wjVg/PLOSOne_formatting_sample_main_body.pdf> and

<https://journals.plos.org/plosone/s/file?id=ba62/PLOSOne_formatting_sample_title_authors_affiliations.pdf>

**ANSWER:**

*We have checked the requirement and hope everything suits your policy.*

2. Please provide additional details regarding participant consent. In the ethics statement in the Methods and online submission information, please ensure that you have specified (1) whether consent was suitably informed and (2) what type you obtained (for instance, written or verbal) and how this information was recorded. If your study included minors under age 18, state whether you obtained consent from parents or guardians. If the need for consent was waived by the ethics committee, please include this information. Please also state the full name of the IRB institute.

**ANSWER:**

*We have obtained written consent, as it is specified in the methods. No minors were included*.

3. We note that Figures 1 and 2 in your submission contain copyrighted images. All PLOS content is published under the Creative Commons Attribution License (CC BY 4.0), which means that the manuscript, images, and Supporting Information files will be freely available online, and any third party is permitted to access, download, copy, distribute, and use these materials in any way, even commercially, with proper attribution. For more information, see our copyright guidelines: <http://journals.plos.org/plosone/s/licenses-and-copyright>.

We require you to either (1) present written permission from the copyright holder to publish these figures specifically under the CC BY 4.0 license, or (2) remove the figures from your submission:

(1) You may seek permission from the original copyright holder of Figures 1 and 2 to publish the content specifically under the CC BY 4.0 license.

We recommend that you contact the original copyright holder with the Content Permission Form (<http://journals.plos.org/plosone/s/file?id=7c09/content-permission-form.pdf>) and the following text:

“I request permission for the open-access journal PLOS ONE to publish XXX under the Creative Commons Attribution License (CCAL) CC BY 4.0 (<http://creativecommons.org/licenses/by/4.0/>). Please be aware that this license allows unrestricted use and distribution, even commercially, by third parties. Please reply and provide explicit written permission to publish XXX under a CC BY license and complete the attached form.”

Please upload the completed Content Permission Form or other proof of granted permissions as an "Other" file with your submission.

In the figure caption of the copyrighted figure, please include the following text: “Reprinted from [ref] under a CC BY license, with permission from [name of publisher], original copyright [original copyright year].”

**ANSWER:**
*We have obtained authorizations. The documents sustaining the rights are attached in the file: figure* authorization.

*We have added “Reprinted from [ref] under a CC BY license, with permission from [name of publisher], original copyright [original copyright year to the caption of copyrighted figure.*

**4**. Thank you for stating the following in the Financial Disclosure section:

"Catherine Hafner
CGR 75659
Edmond J. Safra Foundation.
<https://www.edmondjsafra.org/>
The funders had no role in study design, data collection and analysis, decision to publish, or preparation of the manuscript"

**ANSWER**

*This statement has been added in the Financial disclosure section in the updated Cover letter.*

*Funds were used to pay for salary for one person to transcribe verbatims and fees for one author (JS), as well as for various costs related to the organization of the study.*

*Three of the authors are employed by HUG, while one author: Julie Schneider is an independent graphic designer (at Altitude 436), her time producing materials for the study was financially compensated, without any conflict of interest.*

We note that one or more of the authors are employed by a commercial company: Atelier Julie Schneider Graphic Design, Carouge, Switzerland

**(1)** Please provide an amended Funding Statement declaring this commercial affiliation, as well as a statement regarding the Role of Funders in your study. If the funding organization did not play a role in the study design, data collection and analysis, decision to publish, or preparation of the manuscript and only provided financial support in the form of authors' salaries and/or research materials, please review your statements relating to the author contributions, and ensure you have specifically and accurately indicated the role(s) that these authors had in your study. You can update author roles in the Author Contributions section of the online submission form.

Please also include the following statement within your amended Funding Statement.

“The funder provided support in the form of salaries for authors [insert relevant initials], but did not have any additional role in the study design, data collection and analysis, decision to publish, or preparation of the manuscript. The specific roles of these authors are articulated in the ‘author contributions’ section.”

If your commercial affiliation did play a role in your study, please state and explain this role within your updated Funding Statement.

**ANSWER**

*The following statement is now included in the Funding statement in the cover letter.*

*The funder provided support in the form of salaries for authors JULIE SCHNEIDER, but did not have any additional role in the study design, data collection and analysis, decision to publish, or preparation of the manuscript. The specific roles of these authors are articulated in the ‘author contributions’ section.”*

**(2)** Please also provide an updated Competing Interests Statement declaring this commercial affiliation along with any other relevant declarations relating to employment, consultancy, patents, products in development, or marketed products, etc.

Within your Competing Interests Statement, please confirm that this commercial affiliation does not alter your adherence to all PLOS ONE policies on sharing data and materials by including the following statement: "This does not alter our adherence to  PLOS ONE policies on sharing data and materials.” (as detailed online in our guide for authors [http://journals.plos.org/plosone/s/competing-interests](about:blank)) . If this adherence statement is not accurate and  there are restrictions on sharing of data and/or materials, please state these. Please note that we cannot proceed with consideration of your article until this information has been declared.

Please include both an updated Funding Statement and Competing Interests Statement in your cover letter. We will change the online submission form on your behalf.

Please know it is PLOS ONE policy for corresponding authors to declare, on behalf of all authors, all potential competing interests for the purposes of transparency. PLOS defines a competing interest as anything that interferes with, or could reasonably be perceived as interfering with, the full and objective presentation, peer review, editorial decision-making, or publication of research or non-research articles submitted to one of the journals. Competing interests can be financial or non-financial, professional, or personal. Competing interests can arise in relationship to an organization or another person. Please follow this link to our website for more details on competing interests: [http://journals.plos.org/plosone/s/competing-interests](about:blank)

**ANSWER:**
*This has been added to the cover letter:*

*We confirm you that this affiliation does not not alter our adherence to PLOS ONE policies on sharing data and materials.*

**Reviewer #1**

| 1. When was the study performed? | Between 24.05.2017 et 26.07.2017 |
| --- | --- |
| 2. Focusgroup: what number of participants was your aim? What were criteria for purposive selection (e.g. gender, age, health literacy, experience)? | We wanted to include at least 15 participants of each group (healthcare professionals/patients).  For the healthcare professionals (HP), we included only persons working in an ambulatory setting in Geneva.  For the patients, we wanted to include at least 2 patients of geriatric age. We also wanted to have patients going frequently to medical consultation during the last 5 years (at least 2 persons) and otherwise a majority of patients that have gone between 5-50 times to medical consultations and also include min. 2 patients rarely visiting medical facilities (less than 5 times in the last 5 years). We also wanted to include nonnative French speaking patients > 35% as it is the proportion of migrants living in Switzerland. |
| 3. With respect to the criteria for purposive selection: did you find any indication how the results were influenced by these criteria? Did you look for conflicting evidence? | Unfortunately, our methodology using focus groups for data collection didn’t permit us to analyze our data taking into account the profile of each participant. |
| 4. The definition of VAs is somehow problematic, as you did leave this to the focus group (and later you give direction to the discussion by giving examples). Hence it is not clear what kind of VAs this research addresses. | Our study did not focus on any specific type of VA, which includes different types of media and text association but rather focus on how they were used in practice. Therefore, we decided to explore what this term would mean for our participants through their experience before presenting them different examples. |
| 5. In general, I believe this is not problematic in the context of this paper. As the results show, different types of VA were named during the FGs. |  |
| 6. Please discuss this (also with respect to more modern/complex interventions that include visual material, such as instruction video’s, animations, instruction games). Did you notice any differences in benefits and limitations with respect to the type of VAs? | As the main limitation to use VA cited by healthcare professionals was access, it seemed that easily accessed VA were more used, as material found online (google image for example).  From the patient perspective, it seems that more than the type of VA, the time spent by their healthcare professional on the VA and the explanation, and the possibility to take it home were more important than its type.  Videos were cited and appreciated by patients, but more complex visual aids as serious games were never cited, possibly reflecting that they aren’t used yet in clinical practice. They were also not included in our research scope, as presented material during the FG included only paper based VA. We added this precision in our method. |
| 7. Please include the interview guide in the manuscript (not as supplement). | The interview guide is now table 1. |
| 8. The use of citation seems unbalances (no citation in the first paragraphs, appropriate citations in the second part) | Thank you for your comment. We had to cut them to respect the length of the manuscript. As you notice, this choice unbalances the citations and we added more citations in the first results paragraphs. |
| 9. I believe line ‘117 Professions of healthcare participants’ can be skipped. | This has been removed. |
| •10. Paragraph Identification. o Please support your findings in this paragraph with appropriate citations. | We agree that it is interesting and we have added two verbatims. |
| • 12 Paragraph context o I believe Table 2 might be skipped as the information is well covered within the text. | Thank you for this proposition, we have removed the table 2. |
| o Illustrate the first part ‘context’ with citations | Thank you for this comment, emphasizing the lack of citations in the first half of the results. |
| o Should the text below the Table be part of the Title of Table 3 ‘Purposes and roles of visual tools with cited examples (not exhaustive) and their advantages and constraints of use.’? | Your remark is right, we thought it was an editorial rule. The table has been renamed. |
| o Line 214, page 12. What is the active role (as this is not clear)? Please give an example: The active role of the patient while using “visual tools” is essential but it should also be the case in the process of creation of VA. | This sentence has been added :  “The patients agreed that they also wanted to take an active role, to guide the healthcare practitioner and to create a dialogue while using visual tools.” with a verbatim. |
| • I believe the paragraphs Summary of results and Strengths and limitation should be part of the discussion | Thank you for this comment, this has been changed in the manuscript. |
| Discussion: • Some more limitations might be discussed, such as the interview guide, the very broad scope of the topic (ranging from pictograms to games). | Thanks to your comment, other limitations were added: the broad scope and the single site study. |
| • Discussion please include the following aspects in your discussion o How do the cultural background and patient characteristics such as age, gender and disease characteristics influence the need for VA, and what cultural aspects are most relevant in this context? | We have developed this interesting point as we could, given the limitation of precise analysis which wasn’t the aim of our study. |
| o Co creation: very interesting topic. Is this meant in the context of development of VA, or might this also apply to the use of VA during every single consultation? | Thank you for your comment. Co-creation of VA is meant in both situations (development and use). In our study, patients expressed a will to play an active role during the use of the VA, to guide the HCP during the consultation and to share the use of the VA (this part is better defined now). This topic was also addressed during discussions about designing the VA, this time more by HCP. |
| o How should be accounted for diversity in health literacy in the use and development of VAs? | This is a major issue when using standardized VA. We suggest to develop VA that allow modification/adaptation during the use. For example, several visual types for the same concept allowing the patient to choose his·her preferred one or blank spaces allowing the patient / HCP to add specific notes/drawings. As it is not a finding of our study, we did not add these suggestions in the manuscript. |
| Remark: • Statement concerning data availability: it seems that the underlying data (complete transcriptions, analysis and audio-taped focusgroup interviews) are not available. I do not believe this is needed for this paper. However I believe the statement that “all data underlying the findings described fully available, without restriction, and from the time of publication” is not true. | Thank you for this comment. As asked by the editor, data is now available at GitHUB or upon request to CHafner |

**Reviewer #2** 

| Introduction 1. The introduction should ideally begin with an explanation of the problem that will be addressed in the study, along the lines of “In the medical (…) adapted to patients”, lines 47-49 | We have changed the introduction considering your comment. |
| --- | --- |
| 2. 41-42: The definition of visual aids appears to be incomplete. The description seems to cover ‘visual’ without focussing on the role of ‘aid’ | Thank you for pointing this issue. We completed the definition about the “aid” part, which describe the fact that the image helps to transmit information. We changed our manuscript to define more clearly visual aids. |
| 3. The examples in lines 42-43 are incomplete and not very informative. There are several mentions of ‘.etc’ in the first few pages (lines 38, 42, 52) where it would be more appropriate to present a fuller context. | As said above, the diversity of the expressions used for visual aids made it difficult to summarize it easily. We removed the “etc.” to be more exhaustive, and hope it will make it more clear for our readers. |
| 4. 52: the ‘therefore’ does not follow logically from the sentence before. The authors may want to consider adding something like “It is therefore difficult to draw evidence-based conclusions on what can be considered good practice…” | You are absolutely right. We changed this section to be clearer about the fact that existing studies are focused on the use of a specific VA in research context, and doesn’t really depict real clinical practice.  “Studies focus on a single VA, a single purpose and take place in research context. It is therefore complicated to understand how visual materials can take place into real daily practice with the currently available data.” |
| Methods 5. The described methods generally sound appropriate, but are not tailored to the question on insight into current use of visual aids, for which you would preferably have a wider cross-section. The described method would be better suited to address the question on stakeholders’ perceptions on helpful ways(/barriers) to use visual aids instead. | Thank you for your comment.  We actually consider the current use, but we wanted more information about the experience and the perceptions that a cross-sectional method would have allowed.  It is true that some of the finding could have been similar and with a stronger sample (as for the issue of VA access for example). On the other hand, the risk of VA misunderstanding, the importance of the therapeutic relationship could only have been found with this method. As our further goal is now to create “guidelines” for the use of VA in clinical practice, we believe it was the best method.  We made it more clear how these results will be useful in the text. |
| 6. In addition to this, the focus group guide (supplementary file) does not appear to address practical constraints, which is one of the indicated research questions. | Indeed you are right, we haven’t mentioned the word “constraint” but with the following opened questions, we thought we could identify them :   - What influences the use of VA? - How could images be integrated into daily care? - Which format seems the most suitable?   Indeed, the participants cited constraints (access, time, lack of source). We wanted to identify constraints but with a direct question, maybe we would have had a list of abstract answers, for example budget, time, human ressources etc.. We wanted participants to really focus on their concrete experience or their close future practice. |
| 7. Line 99-100: Please expand on why the separation of patients and HCPs seemed important or how it helped facilitate discussion, e.g. did they feel more comfortable to share their stories? | We anticipated that the perceptions and needs of HCP and patients would be different and that they might be biased by the others’, and that it would help both groups to share their stories without feeling judged. |
| 8. Line 109: Please provide the intercoder reliability for the double-coded section. | CH and JS have double coded 100% of the data on their own and then cross-checked together to reach in the end a 100% consensus. A third opinion was not necessary as consensus was reached. This is why, given our method, we can’t cite an intercoder reliability. |
| 9. Major issue - This section does not meet standards for qualitative reporting. It is generally unclear what was discussed in the interviews and what is interpretation by the authors (e.g. line 214-215: “The active role … creation of VA”). It is also unclear whether findings were mentioned once by a single stakeholder or whether there was group consensus – it would be helpful to add numbers or general indicators (“the group agreed that..”). Quotes should be added, like in section 178-202. The summary of results (283-291) indicates the potential of interesting findings from the interview data, but this is not adequately presented in the results section. | Thank you to remind us the major importance to stay close to our interviews and to avoid interpretation.  We added more citations were they were missing.  Considering the active role, a clarification and a verbatim were added to explicit how data supported this result.  More clarification about what is related to the group or a single participant has been completed. We haven’t found a group consensus on a topic because not all participants expressed themselves every time. We specified if it concerns some participants or only 1. |
| 10. It is great to see the diversity of healthcare professionals included. I am missing the characteristics/demographics of the patient participants. Characteristics such as being a chronic vs new patient, age, literacy levels and cultural background can have great impact on preferences for the use and look of visual aids. | Thank you for this comment. We completed this section with the participants characteristics |
| 11. 125: what do the authors mean with “complete elaborate VA”? | It refers to a VA that is already finished for example, a validated infographic in opposition with a small sketches or an isolated pictogram found on the internet.  Given your helpful comment, we added: (infographics, posters, illustrated sets of cards) |
| 12. 129-135: this is more a methodological consideration re the researchers’ use of language in the interviews than a result to report. | Thank you to underline that this part doesn’t bring any useful data to the manuscript. We removed it. |
| 13. 142+145: the authors make a distinction between ‘visual support’ and ‘visual tool’. However, the language used for this distinction is not very intuitive. If I understand the difference right, I would suggest using ‘stand-alone visual aids’ vs ‘interactive visual aids’. This is in line with what the authors state in line 206 that ‘the main common characteristic of visual tool is their context of use’. | As mentioned in your second comment, we realize that definitions were not clear enough. We have changed the term as it is more appropriate in that way in English. We hope it makes it clearer and more readable for the entire manuscript. |
| 14. 164: The authors list ‘some examples of visual support’ in table 2. This list needs to be extensive, as the aim of the work is to understand how visual aids are being used. | The other reviewer thought that this table should be removed as it is described in the text.  As the complete table is, to our opinion, to big to fit in the article, we chose to add the complete table in the supplementary material. |
| 15. 166, Table 2 – also, 174, Table 3: Were the roles of the visuals and advantages and constraints of use discussed in the interviews, or are these the authors’ interpretations? | All the elements were discussed during the focus groups. The interpretation part is the “practice implications” section, which was modified to be more related to our findings. |
| 16. 166, Table 2: The body of the text talks about the importance of ‘tone’ and ‘frightening pictogram’. The table does not mention emotional effects as a consideration for use. | As reviewer no1 noticed, table 2 doesn’t add any information and actually lacks some. We decided to remove it. |
| 17. 174, Table 3: The authors may want to think about a more specific term for ‘Communication’ as a purpose, as explanation and situation setting are also forms of communication. | You are absolutely right. We precised the term using “Overcoming a communication barrier” |
| 18. 185: Could the authors clarify what they mean with ‘typically visual information’? | We meant information that is already visual (as anatomy). As the term “typically” makes it unclear, we removed it. |
| 19. 230: Could the authors clarify what they mean with “we have not been able to identify a system of how VA are read by patients” | Notes were taken during the FG about how patients read VA (pictures first then text, less detailed picture then more detailed, e.g.) but we couldn’t deduce any reading pattern that would have helped HCP to develop better VA.  We changed the sentence as follow : “we have not been able to identify a systematic pattern of how VA are read by patients. Some patients focused on the text, others on details of the images or the atmosphere of the VA.” |
| 20. 238: Who offered this solution of co-construction? | HCP only did propose the co-construction. We precise it in the text now. |
| 21. 248: Did the patients and healthcare professionals mention it was a good idea to learn a common reference system or do the authors make this claim based on literature? | Interestingly, both patients and HCP mentioned the fact that common reference system exist in other fields (traffic signs, smileys, direction signalization…) and that it could be created in the medical field.  We precise now in the text that both HCP and patients mention this. |
| 22. 258: Who suggested to create a digital database? Seems like a pretty big leap from a conversation about the use of visual aids, and most patients not having much experience with it as the authors describe, to them suggesting the development of a digital database. | Healthcare professionals during the focus groups.  Here is 1 of the 3 verbatims:  HP: “It means we should have a database with all that and we can select what we need (what we want) for the patient. A program, for example, or like that. It takes a long time to make these visual aids when we have to make them ourselves and have to go and get all the info. We spend a lot of time doing that. So if we already had everything and we just had to… select them, put them in a grid, or whatever the medium, it would go faster. (Yeah, okay) And we could make some that are really personalized for the person based on their needs.” (FG1)  However, we agree with you that the development of the database is a further personal project (enthusiastic to us) but it is out of the subject studied so we have removed this part and we thank you for your comment. |
| 23. 265-266: “We have senses… been explicitly stated”. This is not data but the authors’ interpretation. | You are right, and this is the reason why we explicitly stated it. As it’s not a verbatim related and that it might confuse the reader, we removed this sentence. |
| 24. 295-296: Could the authors please elaborate on how their experience has helped to mitigate bias in the research | We have seen some studies where graphic artists create material but they are not the final users. On the other side, healthcare professional don’t have graphical skills to choose pertinent graphical components or adapt them in aesthetic way or to create a common visual language. Working hand in hand in this projects (graphic designers and patients) helped us to realize the gaps on each side and how it is important to stay close to the users’s needs but also to recognize that graphic design is an art that most users don’t handle. |
| 25. 298: If the authors were interested to explore stakeholders’ perceptions on potential uses of visual aids, the statement regarding generalisation might be true to some extent. But findings on how visual aids are being used in a single location are not necessarily generalisable to different healthcare context. The authors should be more realistic and specific about the extent to which their findings are generalisable. | Absolutely. We meant to underline the transversal approach and the fact that “good clinical practices guidelines” could be transversal to different context and healthcare professions.  As it’s not clear, we removed the sentence. |
| Discussion 26. Although there appear to be some interesting findings, this section is confused as well. It presents some data that is not apparent in the results section, e.g. lines 332-33: “several healthcare professionals… for translation purposes” and lines 343-345: “the healthcare professionals… context of the patient.” | Thank you for your attentive reading.  The topic of translation is described in lines 180-183 of the first manuscript. As it may not be clear enough we precised this section (212-215). |
| 27. 307: While I see how the classification of visual aids in practice can be helpful for the research team, I am not convinced that this is a priority finding in the context of use of visual aids and barriers and facilitators in practice. Perhaps the authors can restructure the discussion to place more emphasis on discussing the research questions. | We have restructured the discussion given your pertinent comment and hope it is better. We have really noticed how the classification influences the constraints and can offer some freedom of creation to HP because an interactive VA can be co-constructed with the patient and individualized in a process of oral explanation and dialogue. It switches the paradigm of creating constant sets of materials dedicated for a specific context for a group of patient. |
| 28. 302-303: These sentences seem to contradict each other: “they do not… by their graphic characteristics” vs “…emphasize the importance of aesthetics”, which is shaped by the graphic characteristics. | For us, it is different. The VA can be classified from their graphical characteristics: pictograms, illustration etc… Patients and HP don’t seem to make a distinction between a pictogram and an illustration.  However, aesthetics are influenced by the quality of a drawing or the colors used and the participants were sensitive to those characteristics. |
| 29. 356: The conclusion does not answer the research question of how visual aids are used in ambulatory practice and identifying practical constraints. Instead, the conclusions mainly focus on integrating visual aids in clinical practice, although it does seem to address the question on benefits of visual aids in practice. | Absolutely. You are right to remind us the purpose of this manuscript. We have changed the practice implication and conclusion.  We mentioned more about the benefits we have identified from our study: “valuable tools for explanation, fostering dialogue with the patient, supporting learning and cultivating the therapeutic relationship.”  We agree and have deleted this part. |
| 30. 363-364: I am unsure what this conclusion is based on. |  |
| Practice implication 31. 366-368: This might be a helpful project, but does not follow from the described work and could have been proposed regardless of the specific study findings. |  |
| 32. Spelling/grammar could be improved in places, e.g. leave out ‘have’ in several instances (lines 34, 104, 113…) | We carefully re-read our manuscript before sending our revisions and removed the cited “have”. |
| 33. I would suggest to make the title a more specific: single-site and ambulatory care | You are absolutely right. We changed the title to add your suggestions. |
