# Supplementary material for: Visual aids in ambulatory clinical practice: Experiences, perceptions and needs of patients and healthcare professionals
Source: PLoS One. 2022 Feb 2;17(2):e0263041. doi: 10.1371/journal.pone.0263041 (PMC8809598; doi:10.1371/journal.pone.0263041)
Supplement: Attachment — Submitted filename: Revision letter.docx [file pone.0263041.s002.docx]

Dear Madam, Dear Sir,

Thank you for your careful review of our manuscript. We have addressed our answers to the reviewers’ comments one-by-one below and have outlined changes made in the manuscript. We’ve attached two versions of the revised manuscript, one with marked revisions, and one clean version.

We believe the manuscript has now improved in quality and clarity.

We hope this revised version will meet with your approval and remain at your disposal, should you wish any further information.

Best regards,

Catherine Hafner on behalf of the research team

| 1) Definition of VA (introduction): you seem to focus on non-moving images, also illustrated by your examples. Please add this to your definition. | Thank you for this remark.  We have added this precision.  For the purpose of this article we have defined “visual aids” as all media or formats that are used to give information with the aid of non-moving images (aid to verbal or written information). Images comprise photographs, illustrations, drawings, infographics (method to visually communicate information) or pictograms (Fig 1). |
| --- | --- |
| 2) Methods: include in the manuscript when the interviews were performed. | We have added :  … and took place between 24.05.2017 and 26.07.2017 |
| 3) Approach: purposive selection: please include your criteria for selection of HP as well (including motivation). You should mention some key characteristics of these professionals (experience, gender) as well, and mention if the were distributed well over the focus groups | We have added that we didn’t want to focus on a specific profession.  We have a table listing the variety of professions and we have specified that the distribution of HP in the FG (min 4 different professions).  About the experience and the gender, we cannot give further precise information. |
| 4) Ethics (p6, line 102-103): it is not clear if the proposal was submitted to the Research Ethics Council or not. The authors state: 'full submission was not required'. Does this mean that there was a procedure that the Counsil approved a brief version of the protocol? Or was the protocol not submitted nor judged in any respect at all? | Thanks to your comment, we have completed in the Conduct part:  The research protocol was sent to the Research Ethics Council, which confirmed that in light of our methodology there was no need for a full submission. |
| 5) Minor detail (p7, line 123) authors state that separation of patients SEEMED important. It looks like this WAS CONSIDERED by the authors, and not based on a result of this study. | We understand your comment. We chose to separate patients and HP for the focus groups before they happened. That is why we said it “seemed important”. We still feel it was important given the interaction during the FG but we cannot guess how it would have been with mixed groups.  We have changed the sentence to avoid confusion:  We have separated patients and HP for the focus groups in order to facilitate discussion within the group. |
| 6) Results (p 17, line 287, semantic point): main characteristic of interactive VA was their context of use. I doubt if you can call this a characteristic, if this is part of your definition. It is valuable to read how HP and professionals interact with VA, but this is not be both an essential element of the definition and a characteristic. | Thank you for this very pertinent comment. We have changed this paragraph:  The main common characteristic of the interactive VA is how they are integrated during consultation by the healthcare professional allowing the patient to react, facilitating discussion and interaction. It can help patients to express their choices and the healthcare professional can verify the patient's understanding |
| 7) P20, line 370: please include a citation for 'patients fear time of interaction with HP may be suppressed' as this seems an important point that comes back in the summary of results. | Thank you for your comment.  We have modified the paragraph and included a verbatim:  We have observed that patients are concerned about the time of interaction with the healthcare professional.  Patient: “Because it takes work on the part of the doctors. And when I see that they barely look at the person…they’re stressed out there because there’s the other one waiting (…). At the start, well he gives an explanation (verbal) (…) but then I don’t know if we can still look at the drawing, there indeed… the time…” (FG 5) |
| 8) In my opinion, the abstract does not cover your main findings. You might consider to include the concepts of stand alone and interactive VA, and the purpose of VAs in the abstract. | Thank you for this comment.  We have taken this in account and added: Our study underlines the variety of purposes of use of VA and the different contexts of use allowing the distinction between “stand-alone” VA used out of consultation by patients alone and “interactive” VA used during a consultation enriched by the interaction between HP and patients. (line 30 manuscript without comments) |
